# The molecular mechanism underlying GABAergic dysfunction in nucleus accumbens of depression‐like behaviours in mice

**DOI:** 10.1111/jcmm.14596

**Published:** 2019-08-20

**Authors:** Ke Ma, Hongxiu Zhang, Shiyuan Wang, Huaxin Wang, Yuan Wang, Juhai Liu, Xiaobin Song, Zhenfei Dong, Xiaochun Han, Yanan Zhang, Honglei Li, Abdul Rahaman, Shijun Wang, Zulqarnain Baloch

**Affiliations:** ^1^ College of Traditional Chinese Medicine Shandong University of Traditional Chinese Medicine Jinan China; ^2^ Jinan Center for Disease Control and Prevention Institute of Virology Jinan China; ^3^ School of Food Science and Engineering South China University and Technology Guangzhou China; ^4^ Biomedical Research Center Northwest Minzu University Lanzhou China

**Keywords:** depression, GABA, nucleus accumbens, stress

## Abstract

Depression is the most frequent psychiatric disorder in the world. Recent evidence has shown that stress‐induced GABAergic dysfunction in the nucleus accumbens (NAc) contributed to the pathophysiology of depression. However, the molecular mechanisms underlying these pathological changes remain unclear. In this study, mice were constantly treated with the chronic unpredictable mild stress (CUMS) till showing depression‐like behaviours expression. GABA synthesis, release and uptake in the NAc tissue were assessed by analysing the expression level of genes and proteins of Gad‐1, VGAT and GAT‐3 by qRT‐PCR and Western blotting. The miRNA/mRNA network regulating GABA was constructed based on the bioinformatics prediction software and further validated by dual‐luciferase reporter assay in vitro and qRT‐PCR in vivo, respectively. Our results showed that the expression level of GAT‐3, Gad‐1 and VGAT mRNA and protein significantly decreased in the NAc tissue from CUMS‐induced depression‐like mice than that of control mice. However, miRNA‐144‐3p, miRNA‐879‐5p, miR‐15b‐5p and miRNA‐582‐5p that directly down‐regulated the expression of Gad‐1, VGAT and GAT‐3 were increased. In the mRNA/miRNA regulatory GABA network, Gad‐1 and VGAT were directly regulated by binding seed sequence of miR‐144‐3p, and miR‐15b‐5p, miR‐879‐5p could be served negative post‐regulators by binding to the different sites of VGAT 3′‐UTR. Chronic stress causes the impaired GABA synthesis, release and uptake by up‐regulating miRNAs and down‐regulating mRNAs and proteins, which may reveal the molecular mechanisms for the decreased GABA concentrations in the NAc tissue of CUMS‐induced depression.

## 1 INTRODUCTION

Major depressive disorder (MDD), which is characterized by anhedonia or depressed mood, is a common and debilitating mood disorder in the world.^1^ In terms of its pathogenesis, different reports had suggested that the environmental stresses to the genetically vulnerable individuals were attributed to the depression onset or relapse.^2^ Moreover, the evidence from many clinical trials showed that the early life stress could influence the neural development and lead to the deficiency in brain reward and cognitive circuits, subsequently resulting in the increased risk in depression.^3^ However, the cellular and molecular changes induced by adverse stressor leading to defect in the cognitive and emotional circuits have not yet elucidated.

The hypothesis of GABA dysfunction has long been considered as the important pathological mechanism of depression.^4^, ^5^ The evidence from clinical trials indicated that GABAergic neurotransmission and GABA content were substantially decreased in depressed patients.^6^, ^7^, ^8^ Additionally, GABAergic interneuron is a leading cause of alteration in depressed patients and is beneficial to the increased of self‐focus and cogitation in depressive patients.^9^, ^10^ Our previous electrophysiological study showed that inhibitory synaptic transmission was down‐regulated in NAc GABAergic neurons in the depression model.^11^ The lower GABA content from presynaptic terminals in the NAc tissue may be conferred to the aetiology of chronic unpredictable mild stress (CUMS)‐induced depression. Accumulating evidences indicate that GABA neurotransmission alterations are associated with the pathophysiology of major depression disorder. However, the molecular mechanisms about the reduced levels of GABA in major depression have yet to be fully elucidated.

This study was performed to explore the influences of chronic mild stress on the expression of different GABAergic neurons markers in the mice NAc following CUMS exposure. NAc is considered as a neural interface between motivation and action, which is characteristically disrupted in major depression disorders,^12^, ^13^, ^14^, ^15^ as well as for the depression‐related GABAergic deficits.^16^, ^17^, ^18^ The GABA release associated genes and proteins (Gad‐1, VGAT and GAT‐3) in the NAc tissue were detected by qRT‐PCR and Western blotting (WB). The miRNA/mRNA networks regulating GABA were created based on the bioinformatics analysis and further validated using the method of dual‐luciferase reporter assay in vitro and qRT‐PCR in vivo, respectively. This study could reveal the pathogenic chain of the miRNA/mRNA network regulatory GABA concentrations in the NAc, which is associated with depression‐like behaviours induced by chronic mild stress.

## 2 MATERIALS AND METHODS

### 2.1 Chronic unpredicted mild stress paradigm

The CUMS paradigms experiment was conducted following the previously published protocol.^19^, ^20^ All mice were adapted to daily handling during the week after delivery prior to the experiment**.** Next, mice were randomly divided into control and CUMS group. The control group mice were kept uninterrupted during the treatment period. However, CUMS group mice were treated by a variety of mild stressors (Table S1). Animal ethics committee of Shandong University of Traditional Chinese Medicine approved the protocol used in this study (SDUTCM201805311223).

### 2.2 Behavioural assessments

The behavioural test was performed to evaluate whether the mice following exposure to different stressors presented depression‐like behaviour (anhedonia and behavioural despair). The paradigm of the sucrose preference test (SPT) and novelty suppressed feeding test (NSF) was used to assess the anhedonia behaviour.^21^, ^22^ The behavioural despair was assessed by the forced swimming test (FST) and tail suspension test (TST).^19^, ^23^ All the behavioural experiments were conducted in the sound‐proof behavioural facility with the light cycle.

### 2.3 Quantitative RT‐PCR

After behavioural tests, mice were euthanized and whole NAc tissue was immediately collected as previously described. qRT‐PCR was performed to determine GABAergic neuron‐associated genes and its corresponding miRNAs expression in NAc from CUMS‐induced depression‐like behaviour and control group mice. The primers used for Gad1, VGAT and GAT3, and β‐actin are listed in Table S2. The specific qRT‐PCR procedures referenced our previous study.^19^ The comparative cycle threshold (CT) was used to calculate the relative expression of mRNA and miRNA. All samples were prepared in triplicate.

### 2.4 Western blot analyses

Western blot analyses were performed using standard methods.^24^ The primary antibodies were used as follows: VGAT (A3129, ABclonal Technology), Gad‐1 (ab26116, Abcam), GAT‐3 (AB1574, Minipore) and β‐actin (AC004, ABclonal Technology). The protein signals were visualized using an enhanced chemiluminescence detection system. The optical densities of each band relative to measured values of β‐actin bands were determined using Image‐J software.

### 2.5 Dual‐luciferase reporter assay

The potential target genes of miRNAs were predicted with the use of a bioinformatics database (TargetScan, RNA22, and miRDB). The wild and site‐directed mutation of the detected miRNA‐targeting site in mRNAs 3′‐UTR vector (primer sequences in Table S3) was constructed by following previous protocol.^19^ For the reporter assay, cells were seeded into 24‐well plates one day before transfection. The generated luciferase reporter plasmids, along with the miRNAs mimics or miR‐negative control (miR‐NC), were transfected into cells with the use of Lipofectamine 2000 and then subjected to the dual‐luciferase reporter assay (Promega) for the measurement of the luciferase activity after 48 hours of transfection. Each experiment was performed in the triplicates. The relative rate of firefly luciferase activity to Renilla luciferase activity was calculated.

### 2.6 Statistical analyses

All data were expressed as means ± the SEM. The differences between groups were analysed using two‐tail Student's *t* test and ANOVA *P* values < .05 were considered as statistically significant.

## 3 RESULTS

### 3.1 The behavioural responses to CUMS

**Figure 1:**
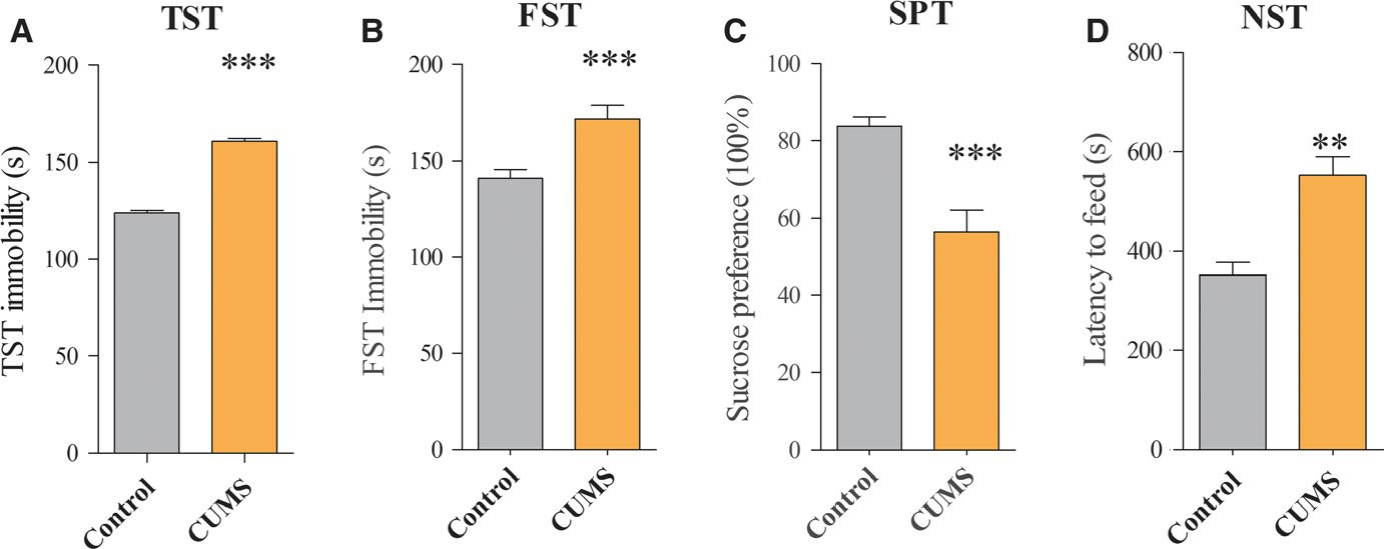
Chronic unpredictable mild stress (CUMS)‐induced depression‐like mice. Following exposure to different stressors for five weeks, the behaviour tests showed the significant decreases significantly increased immobility time in TST (A) and FST (B), as well as exhibited reduced sucrose preference (C) and increased the feed latency in the NST (D) between controls and CUMS‐induced depression‐like mice. The data are expressed as mean ± SEM. n = 10‐14 per group, **P < .01, *** *P* < .001

The depressive behaviours of CUMS‐treated mice were assessed by TST, FST, SPT and NST. Compared to control group, mice exposed to CUMS displayed significant increase in immobility time by TST (123.77 ± 1.39 vs 160.68 ± 1.66, *P* < .001; Figure 1A) and FST (140.77 ± 1.34 vs 171.51 ± 2.1, *P* < .001; Figure 1B). Furthermore, mice exposed to CUMS exhibited significantly lower sucrose preference (83.74 ± 0.68 vs 56.39 ± 1.6, *P* < .001; Figure 1C) and increased the feed latency time (351.3 ± 7.5 vs 555.2 ± 10.76, *P* < .01; Figure 1D) compared to control. Our data indicate that chronic mild stress can induce depression‐like behaviours.

### 3.2 GABA synthesis, release and uptake associated genes expression

**Figure 2:**
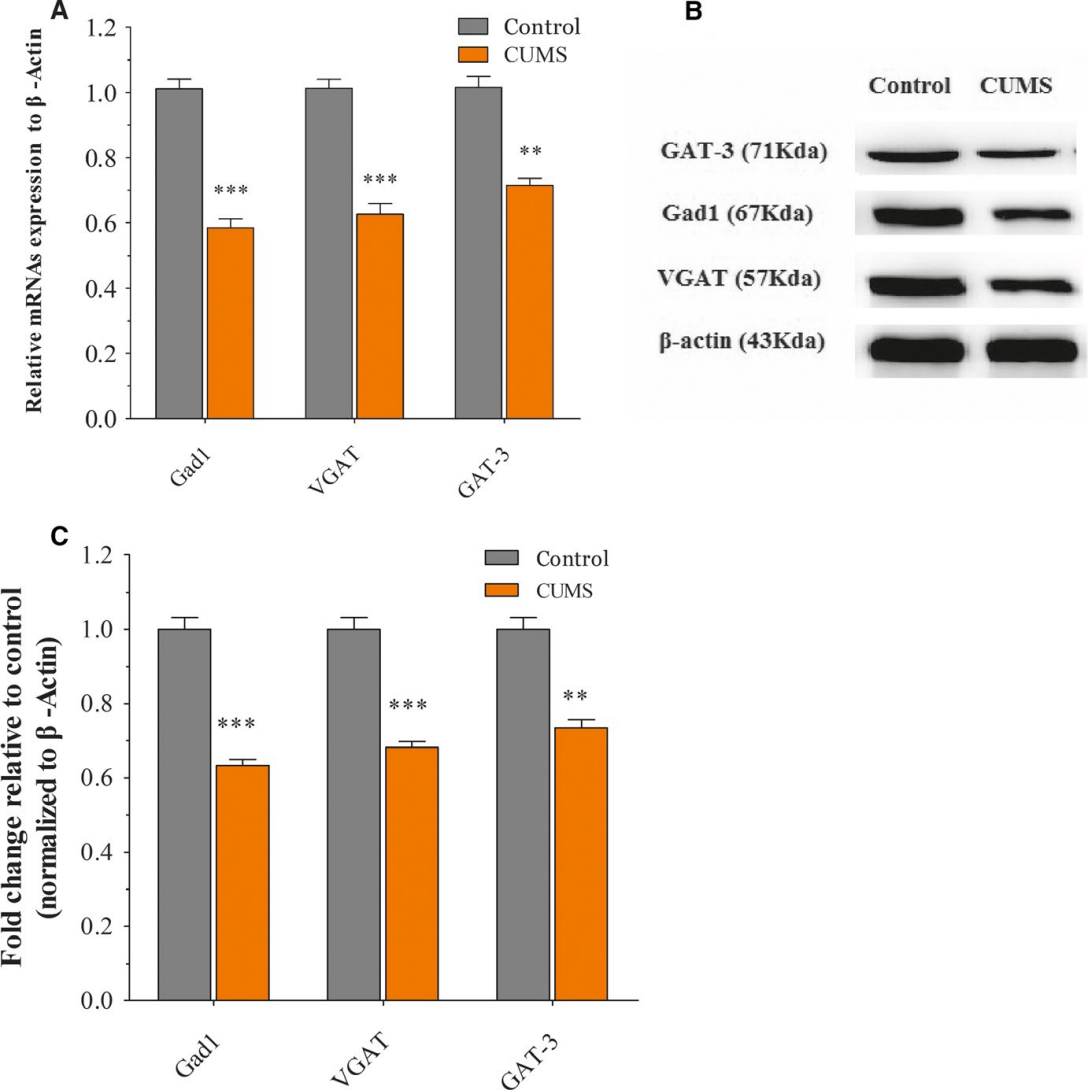
Chronic unpredictable mild stress (CUMS) exposures decrease GABAergic neuron‐associated gene/protein expression level in the NAc tissue. Mice were exposed to CUMS for consecutive five weeks and received behavioural tests. Then, the levels of GABAergic neuron‐associated genes in NAc were determined by qRT‐PCR. A, The relative levels of Gad1, VGAT and GTA3 genes expression in NAc relative to control. B, Representative Western blot images of Gad1, VGAT and GTA3 were shown. C, Statistical analysis of each band relative to measured values of β‐actin bands. All data are presented as mean ± SEM. n = 8‐10 per group, **P < .01, ***P < .001

In CUMS‐induced depression‐like group, the Gad‐1, VGAT and GAT‐3 mRNA expression in the NAc tissue were significantly decreased than that of control mice (both *P* < .01; Figure 2A). Furthermore, the level of Gad‐1, VGAT and GAT‐3 protein has been illustrated in Figure 2B and 2C. The expression level of Gad‐1, VGAT and GAT‐3 proteins was also significantly decreased compared to control mice. There was a significant statistical difference among Gad‐1, VGAT and GAT‐3 proteins expression in the NAc tissue between two groups (both *P* < .01).

### 3.3 The mRNA/miRNA regulatory GABAergic neurons network

**Figure 3:**
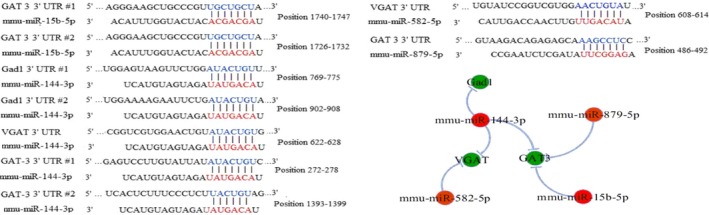
The interaction between mRNAs and miRNAs. miRNAs targeted to mRNAs that encode Gad1, VGAT and GAT‐3 were predicted by using three miRNAs target prediction databases, in which the principle of miRNAs target prediction includes seed match, conservation, free energy and site accessibility. The interactive networks of miRNAs and mRNAs associated with GABA release and uptake are made by using Cytoscape software. Green symbols denote mRNAs that are actually down‐regulated in the NAc tissue. Red symbols denote miRNAs. Their negative regulation of mRNAs by miRNAs is represented by light blue line

Three miRNA targeted‐gene databases (miRDB, RNA22 and TargetScan) were used to predict the VGAT, GAT‐3 and Gad‐1 mRNAs. The 3′‐UTRs of Gad1 (two areas), VGAT (one area) and GAT‐3 (two areas) were targeted by miR‐144‐3p. The 3′‐UTRs of GAT‐3 (two areas) were targeted by miR‐15b‐5p. The 3′‐UTRs of GAT‐3 (one area) were targeted miR‐879‐5p. The 3′UTRs of VGAT (one area) were targeted by miR‐582‐5p (Figure 3). Through bioinformatics analysis, we successfully constructed an epigenetic regulatory network for GABA neuron function.

### 3.4 miRNA‐associated GABA were rise in NAc of CUMS depression mice

**Figure 4:**
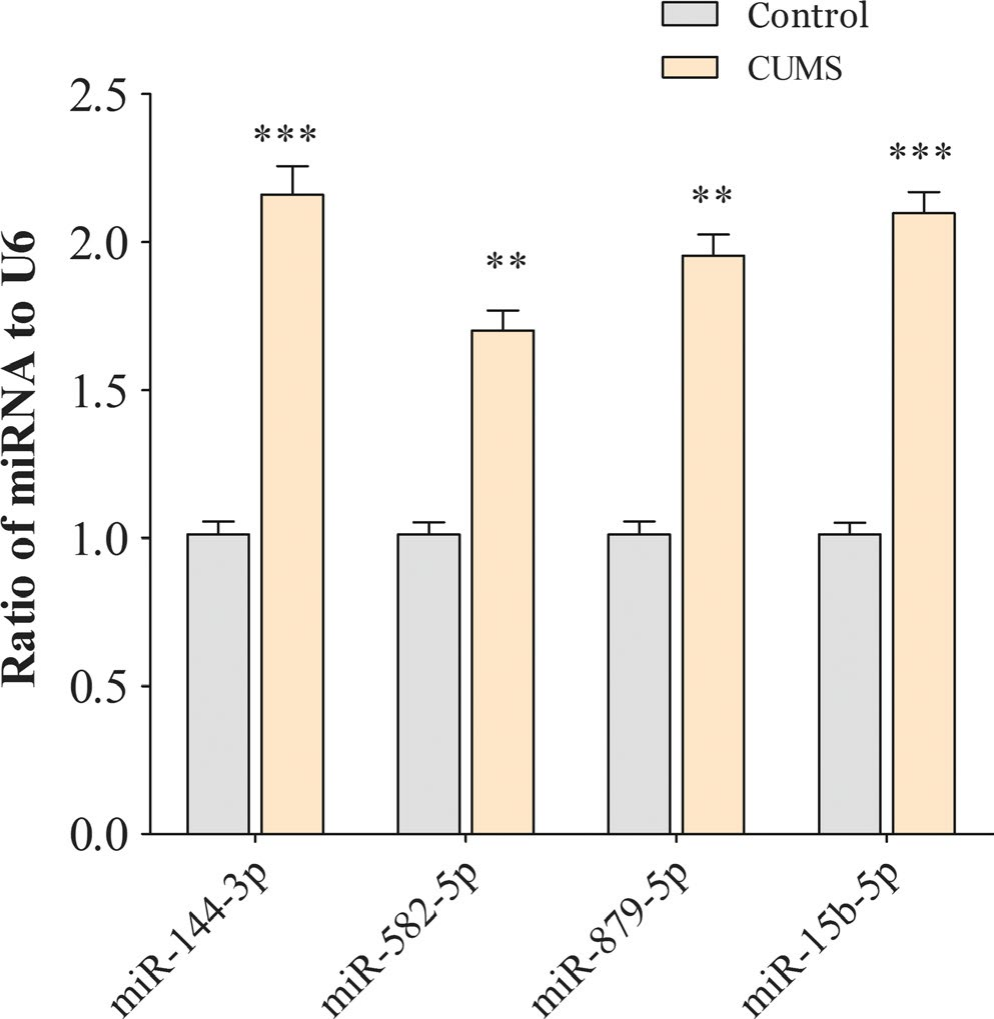
miRNAs of regulating genes associated GABA tone were increased in the NAc tissue of chronic unpredictable mild stress (CUMS) depression‐like mice. qRT‐PCR was used to test the relative expression of miR‐144‐3p, miR‐15b‐5p, miR‐879‐5p and miR‐582‐5p from the controls and CUMS‐induced depression‐like behaviour mice. The relative values for control mice were normalized to be one. Data were expressed as mean ± SEM. n = 10‐14 per group, **P < .01, *** *P* < .001

In order to verify the regulatory network, we quantified four miRNAs among the two groups. Our results showed that levels of miRNAs were significantly increased in the NAc tissue from the CUMS‐induced depression‐like mice than that of control mice (both *P* < .01, Figure 4). The regulatory relationships between the up‐regulated miRNAs and down‐regulated GAT‐3 Gad1 and VGAT mRNAs were presented in Figure 3.

### 3.5 Verification of mRNA/miRNA regulatory GABAergic neurons network in vitro

**Figure 5:**
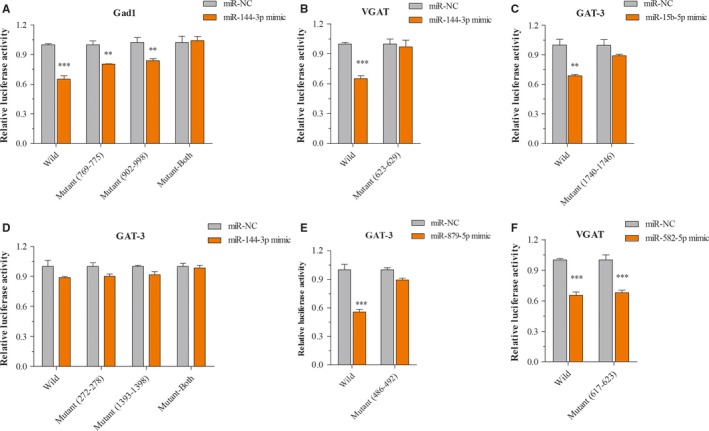
Experimental validation miRNAs targeting genes related to GABA tone. A‐D, The luciferase reporter assay verification association between miR‐144‐3p or miR‐15b‐5p mimic or NC and 3′‐UTR of Gad1, VGAT and GAT‐3 mRNAs. E, The luciferase reporter assay verification association between 3′‐UTR of GAT‐3 mRNA and miR‐879‐5p mimic or negative control. F, The luciferase reporter assay verification association between 3′‐UTR of VGAT mRNA and miR‐582‐5p mimic or negative control. **P < .01, ***P < .001

**Figure 6:**
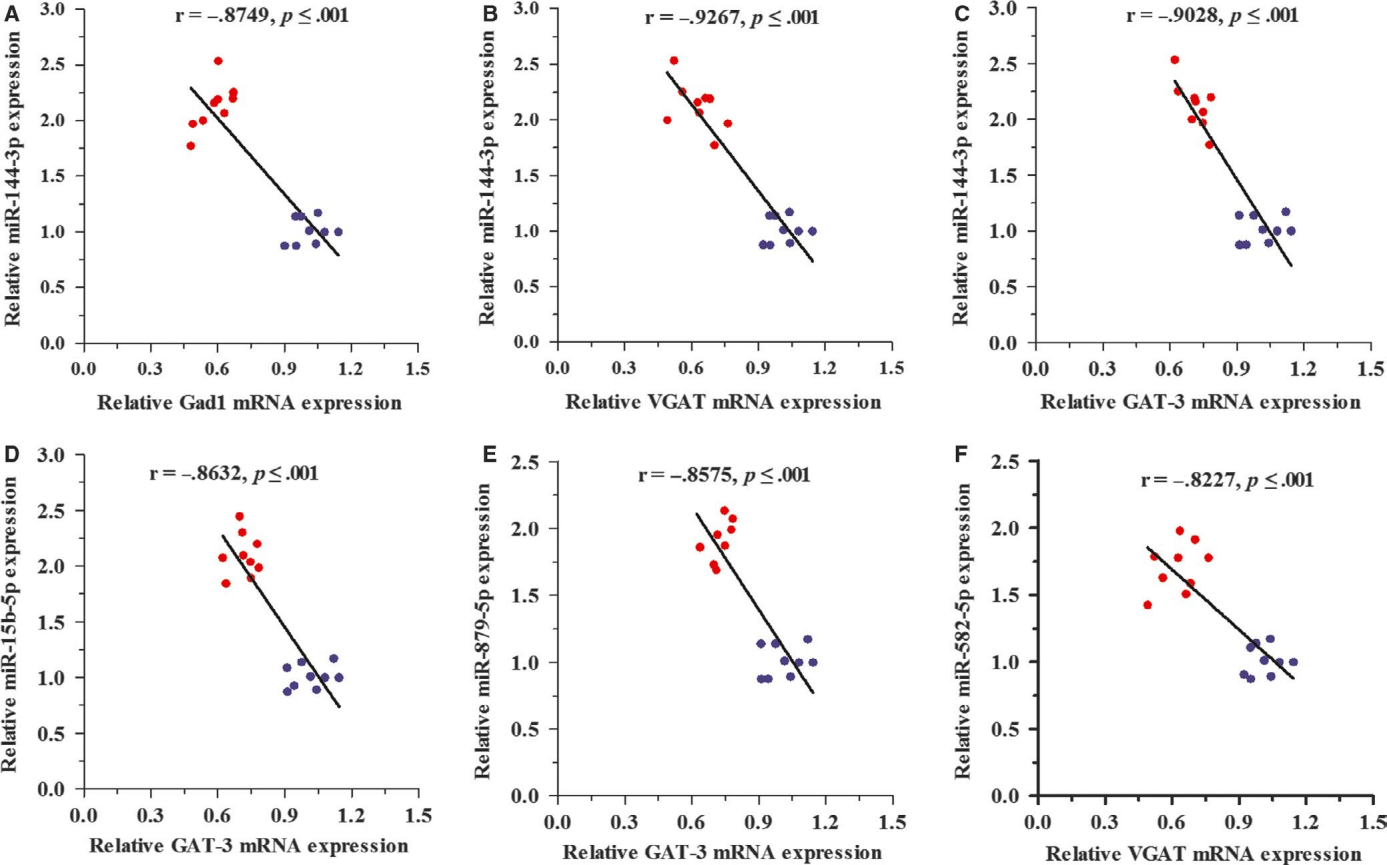
Correlation between miRNAs and their target mRNAs expression in the NAc tissue. The relationships between miRNAs and its corresponding target prediction were assessed by Pearson's correlation coefficients. A, shows the correlation between Gad1 mRNA and miR‐144‐3p expression (*r *= −.8749; *P* < .001). B, shows the correlation between VGAT mRNA and miR‐144‐3p (*r *= −.9267; *P* < .001). C, shows the correlation between GAT‐3 mRNA and miR‐144‐3p (*r* = −.9028; *P* < .001). D, shows the correlation between GAT‐3 mRNA and miR‐15b‐5p (*r* = −.8632; *P* < .001). E, shows the correlation between GAT‐3 mRNA and miR‐879‐5p (*r* = −.8575; *P* < .001). F, shows the correlation between VGAT mRNA and miR‐582‐5p (*r* = −.8227; *P* < .001). The data of qRT‐PCR of miRNAs and mRNAs were analysed in 10‐14 per group. Blue dots indicate congregation of the control group, and red dots indicate congregation of the depression group

Compared to the negative control miRNAs, the miR‐144‐3p (Figure 5A) mimic significantly decreased the luciferase activity by bearing the wild or two separate binding regions mutant in 3′‐UTR of Gad1 (769‐775 and 902‐908). While this suppressive effect was abolished by both mutations in the binding site. Interestingly, miR‐144‐3p also directly regulated VGAT mRNA expression (Figure 5B). Unfortunately, there was no direct interaction between miR‐144‐3p and GAT‐3 (Figure 5D). miR‐15b‐5p and miR‐879‐5p worked as regulators by combing with 3′‐UTR of VGAT mRNA (Figure 5C and 5). The luciferase activity of the VGAT mRNA 3′‐UTR wild‐type was significantly diminished approximately 35% with the introduction of miR‐582‐5p (Figure 6F), while the mutant reporter was able to maintain this suppression effect rather than revising it.

### 3.6 Linear regression analysis of mRNA/miRNA regulatory GABAergic neurons network

To confirm in silico prediction, we performed Pearson's correlation test of mRNA/miRNA regulatory GABAergic neurons network. The linear regression analysis showed that miR‐144‐3p was negatively correlated with the expression of VGAT, Gad1 and GAT‐3 mRNAs in the NAc tissue (Figure 6A‐C). Additionally, there was also a negative correlation between miR‐15b‐5p, miR‐879‐5p and GAT‐3 mRNA expression between the two groups (Figure 6D‐E). While the expression of miR‐582‐5p significantly correlated with VGAT mRNA expression (Figure 6F).

## 4 DISCUSSION

Our previous studies have highlighted the distorted dynamics of neural transmission at the synaptic end of maladapted GABAergic system in the limbic system and been ascribed as the common denominator of major depression.^11^, ^25^ Especially, GABA releases and terminals were significantly decreased in the NAc tissue from the CUMS‐induced depression model (Figure S1). This impairment was caused by the aberrantly expressed level of VGAT, GAT‐3 and Gad‐1 mRNAs or proteins; therefore, it decreased GABA synthesis, release and uptake (Figure 2). In addition, miR‐15b‐5p, miR‐144‐3p and miR‐879‐5p, which were predicted to bind the 3′‐UTR of VGAT and Gad‐1 mRNAs (Figure 3), were significantly up‐regulated (Figure 4). The mRNA/miRNA regulatory GABAergic tone network was assessed by dual‐luciferase assay in vitro (Figure 5) and qRT‐PCR in vivo (Figure 6), respectively.

Recently, several studies have shown that GABA tone substantially decreased in depressive patients or animal model.^6^, ^26^, ^27^, ^28^ GABAergic neurons dysfunction may be as the primary factor for the depression prognosis and pathogenesis.^29^, ^30^, ^31^ Thus, the understanding of molecular and epigenetic mechanisms underlying GABAergic neuron impairment in depression will be useful for the development of novel therapeutics.

In the current study, we investigated the GABAergic marker expression in the NAc to reveal molecular mechanisms underlying reduced GABA release. GABA is synthesized by Gad1/2. While Gad1 is mainly responsible for the GABA synthesis in the brain.^32^ The VGAT biological function was mainly involved in GABA uptake into synaptic vesicles in the presynaptic vesicular membranes.^33^ Furthermore, GABA transporter proteins can either be expressed on neurons or glial cells, which can mediate uptake of GABA from the synaptic cleft.^34^ The consistent results from our study suggested that GABA‐associated mRNAs and proteins expression were decreased in CUMS‐induced depression mice. The decreased level of Gad1 and VGAT expression has already been reported in either depressed patients or depression animal model,^35^, ^36^, ^37^ which are in line with our observations. Interestingly, the decrease GAT3‐expression subsequently decreased GABA uptake might be served as an impaired glial cell indication for depression.^38^ Our finding proved that the production, release and re‐uptake contribute to GABA dysfunction in depression.

miRNAs are a negative regulator of translation by binding to mRNAs 3′‐UTR.^39^ Emerging evidence suggested that miRNAs might play the key role in regulating the process of neurotrophin, serotonergic signalling and synaptic plasticity process.^40^, ^41^, ^42^, ^43^ Our results showed that chronic stress could up‐regulate the levels of miR‐15b‐5p, miR‐144‐3p and miR‐879‐5p expression (Figures 3, 4) as well as down‐regulate the expression of Gad1, VGAT and GAT‐3 genes and proteins, which impaired GABA tone. We validated mRNA/miRNA regulatory GABAergic neurons network by dual‐luciferase assay and qRT‐PCR in vitro or vivo, respectively (Figures 5, 6). At present, the role of miR‐144‐3p in the depressive disorders remains unclear. However, there are a few biological mechanisms that can endorse our finding. miR‐144‐3p has enriched expression and in the brain, as well as in normal and malignant hematopoietic cells and tissues.^44^ Many studies have suggested that miR‐144‐3p was involved in the response to stress, ageing diseases and mood stabilizer treatment.^19^, ^45^, ^46^ In addition, miR‐144‐3p can regulate ataxin 1 (ATXN1) mRNA expression in human cells, and a search of the Genetic Association Database shows that ATXN1 is associated with mental disorders.^47^ miR‐144‐3p‐targeted genes includes Wnt/β‐catenin, Nrf2 and MAKP signalling pathways,^48^, ^49^, ^50^ which have been verified in the physiology of depression.

Our study suggested the potential efficient connection between GABAergic pathway and miR‐144‐3p and miR‐15a/b, which share the same seed region (nucleotides 2‐8) of AGCAGCA, and as such are known as the miR‐15 family. This miRNA family also can target the 3’ UTR of BDNF, cholinergic receptor, muscarinic 1 and methyl‐CpG binding protein 1.^51^, ^52^ All of these targetings have been confirmed in the process of depression pathophysiological mechanism.^53^ These data provided evidence that the miR‐15 family played an important role in the pathogenesis of depression by mediating GABA release and uptake.

In summary, chronic stress leads to the impaired GABAergic deficit by increased miRNAs and corresponding decreased mRNAs and proteins, which reveals sub‐cellular and molecular mechanisms underlying GABAergic dysfunction in the nucleus accumbens of CUMS‐induced depression.

## CONFLICT OF INTEREST

All authors declare no competing interest.

## AUTHORS CONTRIBUTIONS

K Ma, HX Zhang, HJ Zhang and XC Han contributed to experiments and data analyses. Baloch Z and SJ Wang contributed to the project design and paper writing.

## Data Availability

The raw data supporting the conclusions of this manuscript will be made available by the authors, without undue reservation, to any qualified researcher.
