# Supplementary material for: The molecular mechanism underlying GABAergic dysfunction in nucleus accumbens of depression‐like behaviours in mice
Source: J Cell Mol Med. 2019 Aug 20;23(10):7021–8. doi: 10.1111/jcmm.14596 (PMC6787457; doi:10.1111/jcmm.14596)
Supplement: Supplementary file 1 [file JCMM-23-7021-s001.docx]

**Supplemental Table 1: Chronic unpredictable mild stress procedure**

| Stressor | **Mon** | **Tue** | **Wed** | **Thu** | **Fri** | **Sat** | **Sun** |
| --- | --- | --- | --- | --- | --- | --- | --- |
| Food and wather deprivation | 09:00→09:00 |  |  |  |  |  |  |
| Exposure to empty bottles |  | 9:00-11:00 |  |  |  |  |  |
| Soiled cage |  | 11:00→11:00 |  |  | 9:00→21:00 |  |  |
| Restraint |  |  | 11:00-12:00 |  |  |  |  |
| Light/dark succession every 2 h |  |  | 12:00-22:00 |  |  |  |  |
| 45°cage title |  |  |  | 9:00-21:00 |  |  |  |
| Stroboscope |  |  |  | 21:00→9:00 |  |  |  |
| Cold  (4°for 1h) |  |  |  |  | 21:00-22:00 |  |  |
| Cage rotation | 11:00-12:00 |  |  |  |  | 9:00-10:00 |  |
| Wet cage |  |  |  |  |  | 10:00-22:00 |  |
| White noise |  |  |  |  |  |  | 9:00-21:00 |
| Space reduction |  |  |  |  |  |  | 21:00→ |

**Supplemental Table 2. qRT-PCR prime information**

| Gene ID | Symbol | Accession | Prime sequence | Lengths | Tm (°C） |
| --- | --- | --- | --- | --- | --- |
| 243616 | GAT-3 | NM_172890.3 | Forward 5′-GCCACTGGAACAACAAGGT-3′ | 149 | 60 |
|  |  |  | Reverse 5′-TCCGCAGCAGATGAAAAA-3′ |  |  |
| 17196 | VGAT | AK163661.1 | Forward 5′-TGGTCATCGCTTACTGTCTC-3′ | 157 | 60 |
|  |  |  | Reverse 5′-TGCTGCATGTTGCCTTCG-3′ |  |  |
| 14415 | GAD 67 | AF326547.1 | Forward 5'- GGGCTATGTTCCCCTTTATGT-3' | 184 | 60 |
|  |  |  | Reverse 5'-CCTTTCTATGCCGCTGAGT-3' |  |  |
| 11461 | beta (Actb) | NM_007393.3 | Forward 5'- CTACGAGGGCTATGCTCTCC-3' | 145 | 60 |
|  |  |  | Reverse 5'- TTTGATGTCACGCACGATTT-3' |  |  |

**Supplementary Table 3: 3′-untranslated region (UTR) and site-directed mutation prime sequence Gad1, VGAT and GAT-3**

| Gene ID | Symbol | Accession | Prime sequence |
| --- | --- | --- | --- |
| 243616 | GAT-3 | NM_172890.3 | Forward 5′- CCGCTCGAGCCTTGCCTCTTCCCAGTGAT-3′ |
|  |  |  | Reverse ATAAGAATGCGGCCGCAACCACAAGCCTCCCTCTAC |
| 17196 | VGAT | AK163661.1 | Forward 5′- CCGCTCGAGCGAGCTTTAAACACCTCCGG-3′ |
|  |  |  | Reverse 5′- ATAAGAATGCGGCCGCCGCCTTTGTTTCTTCTTTATTTGC-3′ |
| 14415 | Gad1 | AF326547.1 | Forward 5'- CCGCTCGAGTGTCTATCTTTGGGCAGGGG-3' |
|  |  |  | Reverse 5'- ATAAGAATGCGGCCGCAACACTTGTGGGACTGGTCA -3' |
| GAT-3 (272-278) | | Forward 5'- AGTCCTTGTATTATTATTAATCAACTCGTATGCTG-3'  Reverse 5'- CAGCATACGAGTTGATTAATAATAATACAAGGACT-3' | |
| GAT-3 (486-492) | | Forward 5'- GTAAGACAGAGAGCATATATATCCACCATCGTTAGG-3'  Reverse 5'- CCTAACGATGGTGGATATATATGCTCTCTGTCTTAC-3' | |
| GAT-3 (1393-1399) | | Forward 5'- TCACTCTTTCCCTCTAATATAAGTTTCCTAGATTTA-3'  Reverse 5'- TAAATCTAGGAAACTTATATTAGAGGGAAAGAGTGA-3' | |
| GAT-3 (1740-1746) | | Forward 5'- AGGGAAGCTGCCCGTATTATTAACTGCACCTCACCAC-3'  Reverse 5'- GTGGTGAGGTGCAGTTAATAATACGGGCAGCTTCCCT-3' | |
| VGAT (617-623) | | Forward 5'- TGTATCCGGTCGTGGTTAAAATTACTGTGCGTGTGGT-3'  Reverse 5'- ACCACACGCACAGTAATTTTAACCACGACCGGATACA-3' | |
| VGAT (623-629) | | Forward 5'- CGGTCGTGGAACTGTTATTTAAGCGTGTGGTGTGCTC-3'  Reverse 5'- GAGCACACCACACGCTTAAATAACAGTTCCACGACCG-3' | |
| Gad1 (769-775) | | Forward 5'- TGGAGTAAGTTCTGGTATAAAATTATGGTATTTTCGT-3'  Reverse 5'- ACGAAAATACCATAATTTTATACCAGAACTTACTCCA-3' | |
| Gad1 (902-908) | | Forward 5'- TGGAAAAGAATTCTGTATAATTACATAGAGTCATGTT-3'  Reverse 5'- AACATGACTCTATGTAATTATACAGAATTCTTTTCCA-3' | |


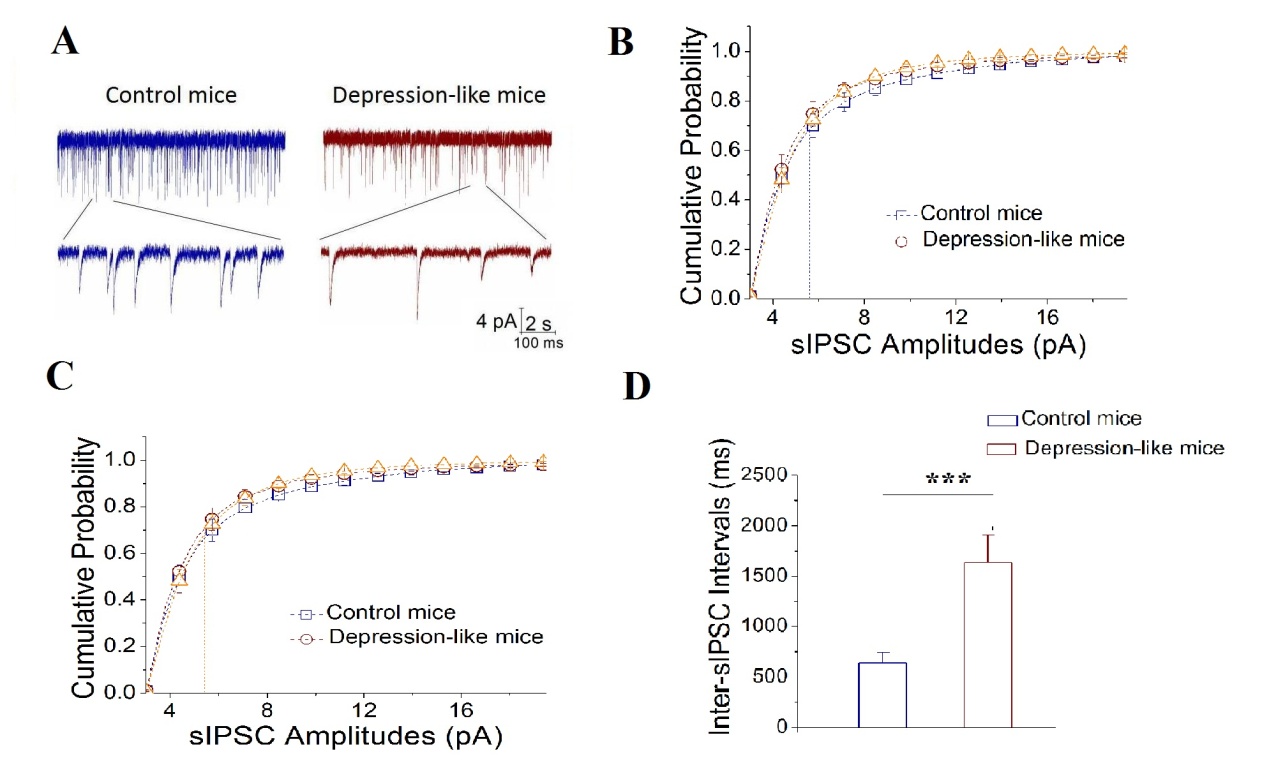


**Supplementary Figure 1: Inhibitory synaptic transmission is downregulated in GABAergic neurons of the nucleus accumbens from CUMS-induced depression mice.** sIPSCs were recorded under voltage-clamp in the brain slices from control and depression-like mice in presence of 10 μM CNQX and 40 µM D-AP5. **A**) Left panel shows sIPSCs from a control mouse, right panel shows sIPSCs from a depression-like mouse. Calibration bars are 4 pA in vertical bar as well as 2 seconds and 100 milliseconds in horizontal. **B**) shows cumulative probability versus sIPSC amplitudes from depression-like mice (red symbols), and control mice (blue). Dash-lines indicate sIPSC amplitudes at cumulative probability to 67% (CP67). **C**) shows cumulative probability versus inter-sIPSC intervals from the depression-like mice and control. Dash-lines indicate sIPSC intervals at CP67 in control and depression-like mice. **D)** shows the inserted fgure shows a comparison of sIPSC intervals at CP67 from the mice of CUMS-induced depression and control.
